# Seed-Assisted Growth of TiO_2_ Nanowires by Thermal Oxidation for Chemical Gas Sensing

**DOI:** 10.3390/nano10050935

**Published:** 2020-05-13

**Authors:** Hashitha M. M. Munasinghe Arachchige, Dario Zappa, Nicola Poli, Nanda Gunawardhana, Nuwan H. Attanayake, Elisabetta Comini

**Affiliations:** 1 SENSOR Laboratory, University of Brescia, Via D. Valotti 9, 25133 Brescia, Italy; dario.zappa@unibs.it (D.Z.); nicola.poli@unibs.it (N.P.); elisabetta.comini@unibs.it (E.C.); 2 Centre for Research and International Relations, Sri Lanka Technological Campus, Padukka 10500, Sri Lanka; nandag@sltc.ac.lk; 3 Department of Chemistry, Temple University, 1901 North 13th Street, Philadelphia, PA 19122, USA; tuf74035@temple.edu

**Keywords:** TiO_2_ nanowires, seed-assisted thermal oxidation, thermal oxidation, TiO_2_ gas sensor, metal oxide nanowires

## Abstract

Herein, we report the catalyst assisted growth of TiO_2_ one-dimensional (1D) nanowires (NWs) on alumina substrates by the thermal oxidation technique. RF magnetron sputtering was used to deposit a thin Ti metallic layer on the alumina substrate, followed by an Au catalytic layer on the Ti metallic one. Thermal oxidation was carried out in an oxygen deficient environment. The optimal thermal growth temperature was 700 °C, in a mixture environment composed by Ar and O_2_. As a comparison, Ti films were also oxidized without the presence of the Au catalyst. However, without the Au catalyst, no growth of nanowires was observed. Furthermore, the effect of the oxidation temperature and the film thickness were also investigated. SEM, TEM, and EDX studies demonstrated the presence of Au nanoparticles on top of the NWs, indicating that the Au catalyst drove the growth process. Raman spectroscopy revealed the Rutile crystalline phase of TiO_2_ NWs. Gas testing measurements were carried out in the presence of a relative humidity of 40%, showing a reversible response to ethanol and H_2_ at various concentrations. Thanks to the moderate temperature and the easiness of the process, the presented synthesis technique is suitable to grow TiO_2_ NWs for many different applications.

## 1. Introduction

The field of one-dimensional (1D) nanomaterial research has witnessed a remarkable growth in its attempt to drive new technologies and improve existing ones. There has been a significant interest over the past decade in 1D nanomaterials owing to their unique physical and chemical properties. These unique properties are impelled by an enhanced surface area and surface electronic properties that can vary enormously from those of their bulk counterparts [1,2]. Among these materials, TiO_2_ 1D nanostructures received enormous attraction in the fields of photo electrochemical water spitting [3], solar cells [4], and optical devices [5] due to their compatible band-edge positions, high resistance to photo corrosion, high photocatalytic activity, lack of toxicity, and low cost [6]. Recently, TiO_2_ 1D nanostructures are gaining a significant interest in the detection of toxic and vapors due to the properties mentioned above [7,8,9]. In particular, TiO_2_ exhibits a great potential for the fabrication of gas sensors due to its high stability at a high working temperature and low cost [10,11,12].

Several attempts have been made to synthetize TiO_2_ nanowires (NWs) including vapor liquid solid (VLS) [13,14,15,16], thermal oxidation [17,18,19,20], hydrothermal [21], sol gel [12,22], pulsed laser deposition [3,23], electrospinning [24,25,26,27], and anodization method [28]. However, wet chemical methods require further cleaning processes and the nanostructures transfer on an appropriate substrate, which increase the cost of the synthesis methods. Therefore, many studies have been conducted to optimize the growth of TiO_2_ NWs via dry physical methods. 

Generally, high-temperature physical synthesis methods lead to the production of contaminant-free high-crystalline TiO_2_ structures in the stable rutile crystalline phase. Lee et al. [13] showed the VLS growth of TiO_2_ nanowires by the thermal evaporation of a Ti source on the alumina substrate, thanks to the deposition of a 500 nm thick Ti buffer layer on top of the Au catalyst. In that work, Ti powder was placed at high temperature inside the furnace and heated to 1050 °C, acting as a source material. Sani et al. [15] demonstrated the VLS growth of TiO_2_ NWs on a 50 nm thick Ti buffer layer by using mixed Ti and graphite powder as a source material to reduce the evaporation temperature of Ti. In general, the thermal evaporation processes reported in the literature always require two steps, the sputtering of a Ti buffer layer and the evaporation of Ti at high temperature [16,29,30,31]. These processes require a high-energy consumption and a complex setup for the nanostructures growth. 

Recent studies focused on the synthesis of TiO_2_ NWs by the thermal oxidation method. This method allows the production of TiO_2_ NWs with a stable rutile phase [32]. The oxidation of a Ti foil in an oxygen environment has been previously studied [17,20,33], and Peng et al. [18] showed that a direct oxidation of the Ti foil was also possible in an organic atmosphere. However, all studies reported so far have been carried out by using Ti foils as a substrate, in the presence of dangerous vapors such as ethanol as an oxidation gas and using an oxidation temperature above 800 °C [34]. 

In this paper, we report for the first time the seed-assisted synthesis of TiO_2_ NWs directly on the alumina substrate by thermal oxidation, starting from a thin Ti layer deposited on the substrate and using only small amounts of oxygen as oxidizing gas. Thanks to the thermal oxidation method, it is possible to avoid the transfer of nanostructures from one substrate to another. This is a great advantage with respect to wet chemical synthesis techniques: The proposed method is highly scalable for a mass production, as it could be easily implemented in industrial processes. The oxidation temperature was reduced compared to the literature, allowing the formation of TiO_2_ NWs on the alumina substrate at only 650 °C. This high-yield method allows the growth of TiO_2_ NWs with an average diameter of 20–40 nm, with a length of several micrometers. Furthermore, these TiO_2_ NWs show a reversible response to ethanol and H_2_ when integrated in chemical sensing devices.

## 2. Materials and Methods 

Alumina substrates (2 × 2 mm, Kyocera, Japan, 99.9% purity) were used to deposit and grow TiO_2_ NWs. These substrates were ultrasonically cleaned for 15 min in acetone to remove dust particles, and then dried in a synthetic air flow. A thin metallic Ti layer (50, 100, or 200 nm) was deposited by RF magnetron sputtering (75 W argon plasma, chamber pressure 5.0 mTorr, and 300 °C with a deposition time of 25, 50, or 100 min). Subsequently, a deposition of a thin Au layer was performed on the Ti surface by RF magnetron sputtering (75 W argon plasma, chamber pressure 5.0 mTorr, room temperature), resulting in an agglomeration of nanoparticles (2–5 nm) acting as seed catalysts for the nanostructures growth. Finally, samples were placed in a homemade thermal oxidation chamber and were oxidized. The internal pressure of the chamber was maintained at 10^−1^ mbar during the oxidation, which was carried out in a temperature range between 600–750 °C for 2–4 h in a mixed gas flow of O_2_ (1–20 Standard cubic centimeters per minute (SCCM)) and argon (100 SCCM). Table 1 collects all experimental parameters controlled during the synthesis process.

The surface morphology of TiO_2_ NWs was investigated by a field emission scanning electron microscope (FE-SEM, LEO 1525, Zeiss) operating at 3–5 kV. Transmission electron microscopy (TEM) images were obtained using a JEOL JEM-1400 microscope operating at 120 kV. Raman measurements were performed using a Horiba Jobin Yvon Labram HR800 Evolution confocal Raman spectrometer with a 532 nm laser excitation, Olympus MPlan N 100× microscope objective able to focus the excitation light to ~1 µm spot, and 1800 g/mm grating providing ~2 cm^−1^ spectral resolution.

**Figure 1:**
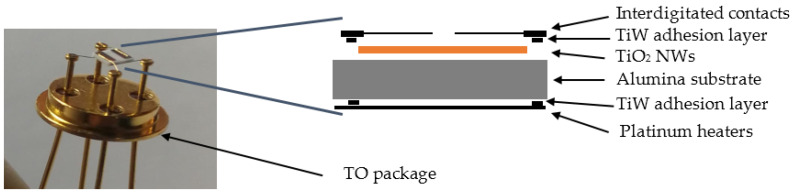
Schematic of the conductometric device fabricated.

After the nanostructures synthesis and characterization, chemical sensing devices were fabricated as shown in Figure 1. For the functional characterization of the sensing devices performances, we used the same alumina substrates covered by TiO_2_ NWs as active transducers. A TiW adhesion layer and Pt interdigitated electrodes (IDE) were deposited by DC magnetron sputtering on top of TiO_2_ NWs (75 W argon plasma, 7 SCCM argon flow, 5.0 mTorr pressure at 300 °C). The TiW adhesion layer was placed between the platinum IDE and the alumina substrate to improve the mechanical adhesion of the stacked structure. 

Metal oxide interactions with target gas molecules are thermally activated. Therefore, platinum heaters were fabricated on the backside of the alumina substrate using the same conditions used for the electrodes. Finally, substrates were mounted on transistor-outline (TO-39) packages by using electro-soldered gold wires. The sensors conductometric responses were tested in a stainless steel chamber (1 L volume) located inside a climatic chamber (Angelantoni, Italy, model MTC 120) with temperature set at 20 °C. Humidified air was produced by flowing the dry air through a Drechsel bottle, held in a thermostatic bath at 25 °C. Four different sensors were mounted inside the test chamber, which is able to measure the performances of the sensors simultaneously. A fixed voltage (1 V) was applied to the sensor active films and the total gas flow was set to 200 SCCM with 40% relative humidity (RH).

Fabricated devices were tested towards ethanol, acetone, carbon monoxide, and hydrogen at 300, 400, and 500 °C. Prior to the introduction of the target gas, samples were stabilized for 10 h at each temperature. Sensors were exposed to the selected gas concentration for 20 min, and then the synthetic air flow was restored to recover the baseline signal. The devices response was calculated from the variation of their electrical conductance, using the following formulae for oxidizing and reducing gases, respectively.

(1) $$Response=\frac{R_{Gas}-R_{Air}}{R_{Air}}=\frac{G_{Air}-G_{Gas}}{G_{Gas}}$$

$R_{Air}$ and $G_{Air}$ are the base line resistance/conductance of the sensor and $R_{Gas}$ and $G_{Gas}$ are the resistance/conductance of the sensor in the presence of the target gas. 

## 3. Results

### 3.1. Morphological and Structural Characterization

**Figure 2:**
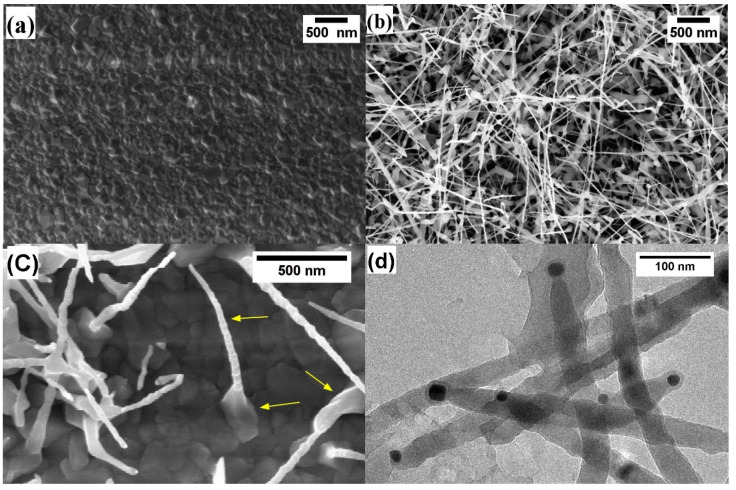
SEM plain views of TiO_2_ nanowires (NWs) growth at 700 °C with 10 SCCM oxygen and 100 SCCM argon flow for 3 h, (**a**) without gold catalyst and (**b**) with gold catalyst. (**c**) TiO_2_ NWs with narrow needle-like top and wide grains at the bottom. (**d**) TEM image of the TiO_2_ nanowires.

As pointed out in the previous section, TiO_2_ NWs were grown on the alumina substrate by thermal oxidation in an oxygen deficient environment. Figure 2 illustrates the SEM and TEM images of Ti (200 nm thickness) samples, grown at 700 °C with 10 SCCM oxygen and 100 SCCM argon flow for 3 h. Figure 2a shows the pure Ti metal growth at 700 °C in the mixture of Ar/O_2_. Many dense polycrystalline grains were formed during the oxidation of the Ti metal, but these polycrystalline grains did not show any uniform orientation in the growth direction. Figure 2b shows the SEM plain-view images of the Au/Ti growth at similar conditions. The results indicate that the Au layer has a strong influence on the NWs growth, confirming that the growth of TiO_2_ NWs is driven by Au nanoparticles (NPs) [35]. Further investigations reveal that these NWs have two dominant segments (Figure 2c). These segments of the NWs are composed of a narrow needle-like top and wide grain at the bottom. 

**Figure 3:**
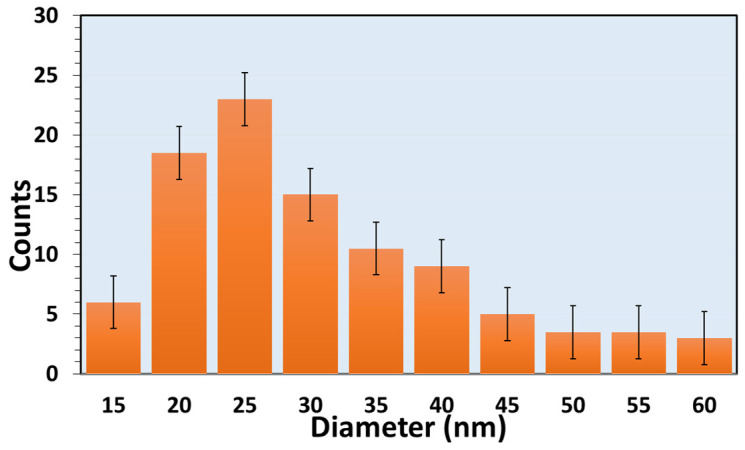
Diameter distribution of the thermally oxidized TiO_2_ nanowires growth at 700 °C with 10 SCCM oxygen and 100 SCCM argon flow for 3 h.

Figure 2d shows the TEM image of some TiO_2_ NWs, removed from the alumina substrate and dispersed on a carbon grid. TEM images clearly illustrate the presence of Au nanoparticles on top of the NWs, further confirming that Au nanoparticles have a fundamental effect on the NWs growth. NWs exhibit a quite uniform diameter and have a neck-like narrow-end close to the Au nanoparticles. Figure 3 reports the diameter distribution of the TiO_2_ NWs. The average diameter of TiO_2_ NWs was 20–40 nm while the length was several micrometers. In the end, this growth method resulted in being scalable and reproducible.

**Figure 4:**
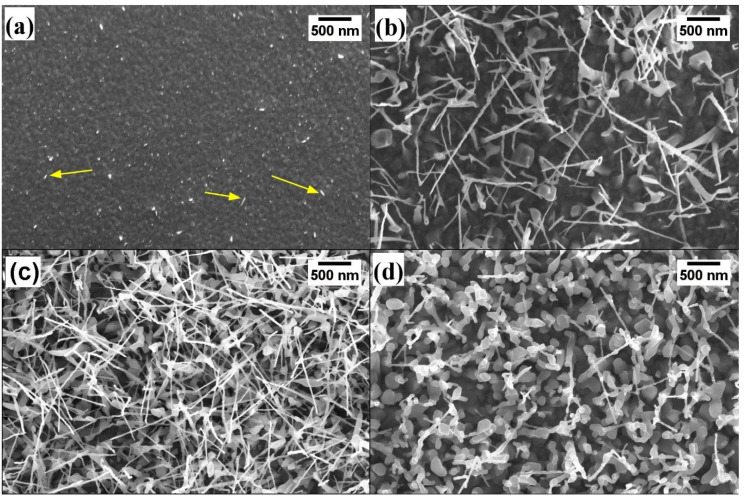
SEM view of TiO_2_ nanowires grown at different temperatures: (**a**) 600, (**b**) 650, (**c**) 700, and (**d**) 750 °C. Oxidation time was 3 h and the oxygen and Ar flow was set 10 and 100 SCCM, respectively.

Figure 4 reports the SEM images of Au/Ti samples grown in the presence of 10 SCCM oxygen and 100 SCCM argon flow for 3 h at different temperatures. At 600 °C (Figure 4a), TiO_2_ grains are well distinguished on the surface but NWs appear only in a few spots with a nonuniform distribution and very short length. A huge difference was observed by increasing the temperature to 650 °C. Figure 4b shows the long and thin NWs grown at this temperature. However, no significant growth improvements can be obtained by increasing the oxidation time to 4 h at 650 °C. In addition, wide grains are observed beneath the NWs. On the contrary, dense, long, and thin NWs were formed when the oxidation temperature was increased to 700 °C (Figure 4c). Early studies demonstrate that the Ti diffusion temperature required to form nanowires is above 800 °C (in Ti foils) [20,32]. Nevertheless, we observed that this temperature decreases to 650 °C in the presence of Au nanoparticles. At 750 °C, instead, thick nanorod-like structures can be observed at the bottom of the NWs, confirming that 700 °C is the optimum temperature for TiO_2_ NWs growth.

**Figure 5:**
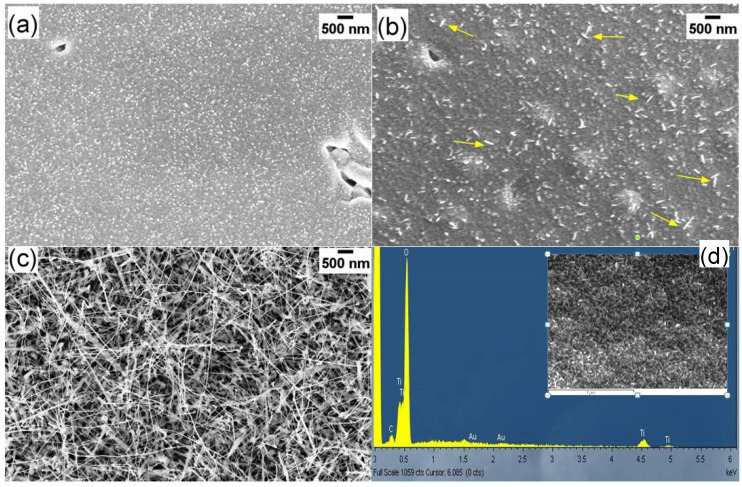
SEM view of TiO_2_ nanowires grown from different film thicknesses: (**a**) 50, (**b**) 100, (**c**) 200 nm at 700 °C, (**d**) EDX spectra of the 200 nm thick TiO_2_ NWs sample. Oxygen flow and Ar flow were set at 10 and 100 SCCM, respectively.

Figure 5 reports the effect of the layer thickness on the synthesis of TiO_2_ nanostructures, which is significant. It is not possible to observe any TiO_2_ NWs if the thickness of the Ti metal layer is 50 nm (Figure 5a). The NWs growth, instead, is well visible on 200 nm thickness films (Figure 5c), while very short and few NWs can be seen when the thickness is decreased to 100 nm (Figure 5b). Figure 5d shows the EDX spectra of thermally oxidized 200 nm film, revealing the presence of Au. In fact, minor Au peaks can be seen at 2.3 KeV, confirming the presence of Au after the oxidation process on the nanowires tip. 

No significant changes were observed by varying the oxygen flow from 1 to 20 SCCM. Finally, we studied the effect of oxidation time also. Thin and long NWs were observed when the oxidation time was set to 3 h, and further oxidation results in the growth of flake-like structures. Thanks to the previous considerations, optimum conditions for the synthesis of TiO_2_ NWs were identified. The optimal Ti layer thickness was 200 nm, the gas flow was 10 SCCM of oxygen and 100 SCCM of argon, 3 h of deposition, and an oxidation temperature of 700 °C. 

**Figure 6:**
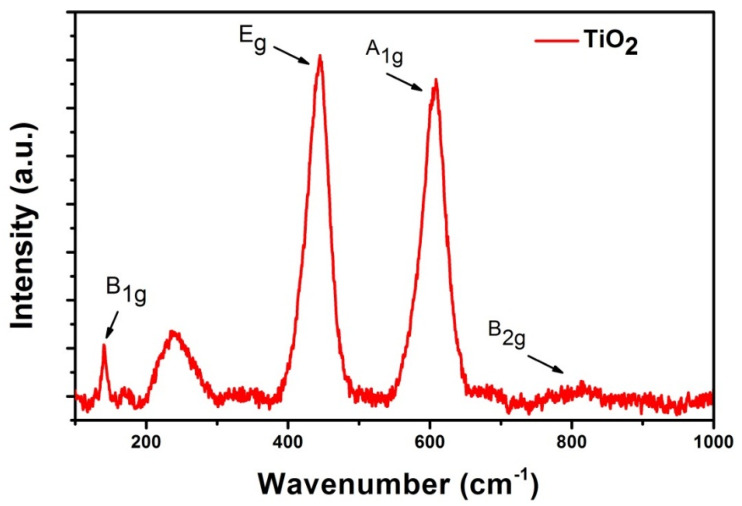
Raman spectrum of TiO_2_ nanowires growth at 700 °C, 10 SCCM oxygen, and 100 SCCM argon flow for 3 h.

Apart from the SEM and TEM analysis, TiO_2_ NWs were also analyzed by Raman spectroscopy (Figure 6), confirming the rutile crystalline structure. TiO_2_ active vibrations were located at 142 cm^−1^ (B_1g_), 238 cm^−1^ (broad band), and 444 cm^−1^ (E_g_), 624 cm^−1^ (A_1g_), and 824 cm^−1^ (B_2g_) [36,37,38]. The broad Raman band at 238 cm^−1^ could be due to the second-order scattering or disorder effects. Galstyan et al. reported the phase transition from anatase to rutile at the annealing temperature of 500 °C [3]. Therefore, our results are in good agreement with the theoretical analysis and published data, confirming that the TiO_2_ NWs prepared by the proposed method have a rutile phase without any significant impurities [39,40].

### 3.2. Growth Mechanism

**Figure 7:**
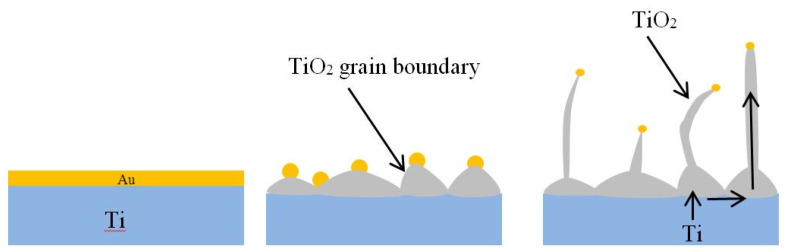
Effect of the Au catalyst on the growth of TiO_2_ NWs.

SEM, EDX, and TEM images confirmed the presence of Au nanoparticles on the tip of the TiO_2_ NWs, showing that the process may be similar to the vapor liquid solid (VLS). However, in our case there is no external Ti source: The Ti source is directly the sputtered Ti layer. Ti reacts with oxygen forming a TiO_2_ film when exposed to an oxygen environment. Then, the oxygen further diffuses through the oxide layer to the metal interface, causing a polycrystalline grain growth (Figure 7). Previous studies have shown that Ti atoms diffuse from the Ti layer to Au, forming intermetallic phases TiAu_4_, TiAu_2_, TiAu, and Ti_3_Au [41]. The diffusion can occur in a local gradient of chemical potentials in the presence of Au NPs [32,42]. The presence of the Au catalyst is of utmost importance in the synthesis of Ti NWs, as Au promotes preferential nucleation of TiO_2_ on its surface and it also suppresses TiO_2_ nucleation on other sites of the substrate. The presence of oxygen in the atmosphere promotes the elongated growth of TiO_2_ along the principal growth direction. The wider base and the tapering shape of the NWs can be explained based on the diffusion of Ti from the sputtered layer during the growth. This base can be clearly observed when the oxidation time is longer than 3 h. Figure 7 shows that few NWs cause a change of direction with very sharp edges. This is due to the extended defects in the crystallographic structure of the NWs [32]. These defects can lead to an abrupt change of crystal structure orientation in NWs, which results in a sharp change of the main growth direction.

### 3.3. Gas Sensing 

**Figure 8:**
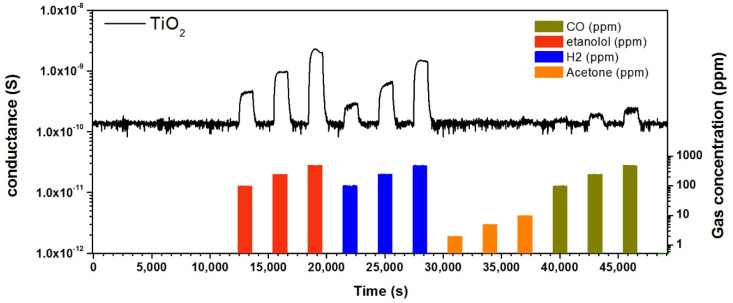
Dynamic response of TiO_2_ NWs toward ethanol (100, 250, 500 ppm), H_2_ (100, 250, 500 ppm), acetone (2, 5, 10 ppm), and CO (100, 250, 500 ppm) at 400 °C. RH = 40%, with an applied voltage equal to 1 V.

**Figure 9:**
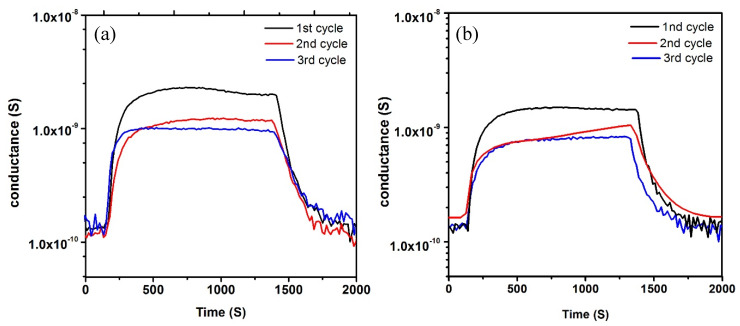
The repeatability of TiO_2_ NWs sensors at 400 °C exposed to (**a**) ethanol (500 ppm) (**b**) hydrogen (500 ppm). RH = 40%, with an applied voltage equal to 1 V.

Optimized samples (Ti layer thinness 200 nm, 10 SCCM of oxygen flow, 3 h, and oxidation temperature at 700 °C) were used to fabricate chemical sensing devices and for their characterization in the presence of target chemical compounds. More specifically, we have investigated the conductometric response of the fabricated material in the presence of different chemical species such as ethanol, acetone, H_2_, and CO in a wide temperature range (300–500 °C), in order to understand the sensing behavior. Figure 8 shows the dynamic response of TiO_2_ NWs towards ethanol, acetone, H_2_, and CO at a working temperature of 400 °C. The electrical conductance increases when introducing a reducing gas and increases with the gas concentration. As the synthetic air flow is restored, the conductance decreases recovering the baseline value. This behavior is typical of n-type semiconductors. At the operating temperature of 300 °C and below, the conductance is very low and noisy. Moreover, the response is very low toward the investigated chemical compounds. Figure 9 reports the short-term stability of the TiO_2_ NWs based gas sensors, resulting in the exposure of the same gas concentrations over a short period. A very small change in the baseline conductance was observed within the different cycles. On the other hand, the sensor response towards ethanol and hydrogen slightly decreased after the first measurements, probably due to thermal stabilization over the measuring period. The response was stable after the first exposure to the target analyte. 

**Figure 10:**
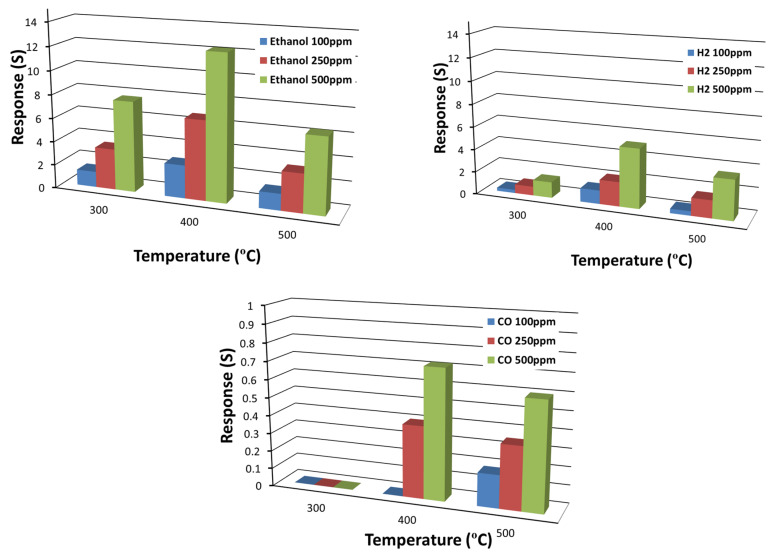
Response towards H_2_, ethanol, and CO in different working temperatures. RH = 40% at 20 °C with applied voltage equal to 1 V.

Figure 10 indicates the temperature dependence of the device performances in terms of response. Overall, TiO_2_ NWs show good responses to ethanol and H_2_. The response toward hydrogen is maximum at 400 °C, while it decreases at higher temperatures. The response of TiO_2_ NWs towards ethanol is high at all working temperatures; nevertheless, also for ethanol detection the optimal working temperature is 400 °C. The temperature has a high impact on the response for ethanol, it increases more than two times from 300 to 400 °C. A low response was observed for CO even at 500 °C, less than one at any operating temperature. We have also tested TiO_2_ NWs toward other chemical compounds such as acetone, but the response was negligible.

**Figure 11:**
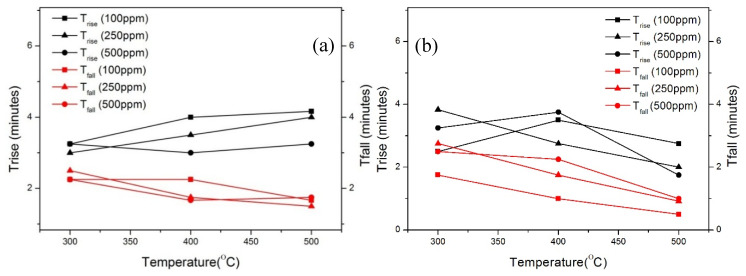
Response time and recovery time of the TiO_2_ NWs in the temperature range of 300–500 °C to (**a**) ethanol (**b**) hydrogen.

The response time and recovery time could be defined as the time to reach 90% of the steady state of G_gas_ and G_air_, respectively. Figure 11 shows the influence of the temperature and gas concentrations towards both response time and recovery time. The response time for ethanol is about 2 to 4 min at all temperatures ranging from 300 to 500 °C (Figure 11a). In addition, the response time at 300 °C is lower compared to 500 °C. The recovery time is more than 2 min at 300 °C and less than one minute at 400 °C, indicating that these sensors have a fast recovery. The response time and recovery time is less than 4 min for hydrogen and it decreases for higher working temperatures. Moreover, Figure 11 shows that the gas concentration has no significant impact on the response time and recovery times. Considering the volume of the test chamber (1 L) and the gas flow used (200 SCCM), it takes approximately 15 min to have a complete atmosphere change inside the measuring chamber, by pumping three times the volume of the chamber. Nevertheless, from Figure 11 we may observe that the devices start responding before this complete atmosphere change (<4 min). Therefore, we can suppose that the response and recovery times are overestimated and that the sensing devices are much faster than reported.

**Figure 12:**
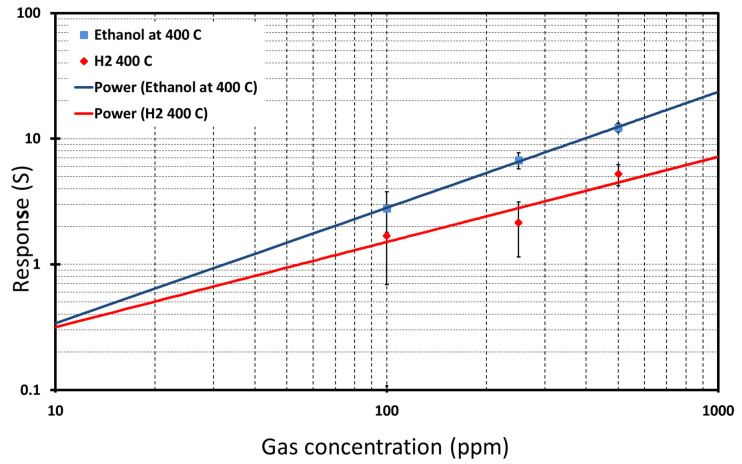
Calibration curves of TiO_2_ NWs reporting the response versus gas concentration at optimal temperatures and relative humidity of 40% at 20 °C.

Figure 12 shows the estimated calibration curve and power fitting for TiO_2_ NWs for ethanol and H_2_ at optimum working temperatures. The experimental data can be fitted by a typical power relation for metal oxide conductometric sensors:

(2) $$\mathrm{Response}=A{\left[\mathrm{Gas}\;\mathrm{concentration}\right]}^{B}$$

where A and B are constants typical of the gas sensing material and stoichiometry of the involved reactions. The detection limit was calculated by taking one as a threshold response to ethanol and H_2_. These TiO_2_ NW detection limits for ethanol and H_2_ were identified as 30 and 90 ppm, respectively. The detection limit for ethanol is lower compared to H_2_. Table 2 summarizes the values of A and B coefficients and the detection limits of TiO_2_ NWs. 

Table 3 reports the literature data on gas sensing properties of TiO_2_ NWs and nanobelts, synthesized by various methods, towards ethanol and hydrogen. TiO_2_ nanobelts and nanowires synthetized via the hydrothermal technique exhibit good responses to ethanol at 350–400 °C in the presence of humidity [43,44]. However, most of the other results show a good response only in the absence of humidity [45,46,47]. Moreover, as a hydrogen gas sensing material, it shows a noble response to 500 PPM at 400 °C by comparing with other results [48,49]. However, most of the studies have been carried out in a high concentration of hydrogen or without the humidity environment [50,51,52]. From these results, it can be concluded that TiO_2_ NWs grown by thermal oxidation show a high response to ethanol and H_2_ even compared to state-of-the-art results. 

### 3.4. Gas Sensing Mechanism

The sensing properties of the TiO_2_ NWs are based on the change of the electrical resistance due to the adsorption and desorption of the chemical species from the surface of the sensing material. This mechanism has been adopted for many semiconductor metal oxides and it was described elsewhere in detail [53]. When the sensor is exposed to air, oxygen molecules adsorb on the TiO_2_ surface, forming oxygen ions, and capturing electrons from its conduction band, leading to an increase of the metal oxide electrical resistance. The reaction kinetics are shown by the following reactions [54]:

(3) $$O_{2\left(gas\right)}\to O_{2\left(ads\right)}$$

(4) $$O_{2\left(ads\right)}+e\to O_{2\left(ads\right)}^{-}$$

(5) $$O_{2\left(ads\right)}^{-}+e\to 2O_{\left(ads\right)}^{-}$$

(6) $$O_{2}^{-}+e\to O_{\left(ads\right)}^{2-}$$

Some oxygen atoms may diffuse and spill over from the Au nanoparticles onto TiO_2_ NWs, capturing electrons from the conduction band. This mechanism increases oxygen adsorption, resulting in an enhancement of NWs interaction with the target gas molecules. When the sensors are exposed to ethanol and H_2_, gas molecules may be chemisorbed and react with adsorbed oxygen species to form H_2_O. This will result in the release of trapped electrons back to TiO_2_ with a decrease in the sensor resistance. Possible reactions are as follows:

(7) $$C_{2}H_{5}OH_{\left(ads\right)}+O_{\left(ads\right)}^{-}\to CH_{3}CHO_{\left(ads\right)}+H_{2}O+e^{-}$$

(8) $$H_{2}+O_{\left(\mathrm{ads}\right)}^{-}\to H_{2}O_{\left(\mathrm{gas}\right)}+e^{-}$$

## 4. Conclusions

In conclusion, TiO_2_ NWs were grown on the alumina substrate by the seed assisted thermal oxidation method. RF magnetron sputtering was used to deposit Ti and Au layers. Thermal oxidation was carried out in an oxygen deficient environment. Morphological characterization revealed the presence of Au nanoparticles on top of the NWs, indicating that the Au catalyst drove the growth process. Raman spectroscopy confirmed the rutile crystalline phase of the material. All the morphological investigations confirmed that the oxidation temperature, film thickness, and oxidation time play a crucial role in the growth of TiO_2_ NWs. Moreover, gas sensing measurements showed the ability of TiO_2_ NWs to detect ethanol and H_2_ at an optimal temperature of 400 °C. The detection limits resulted below 50 and 100 ppm for ethanol and H_2_, respectively. These results demonstrate that TiO_2_ NWs are good candidates for the fabrication of a chemical/gas sensing device. Moreover, this synthesis technique may be adopted further to grow TiO_2_ NWs on different transducers for their employment in applications such as photo electrochemical water spitting and solar cells.

## Figures and Tables

**Table 1:** Experimental condition tested for the synthesis of TiO_2_ NWs.

Parameter	Condition
Ti layer thickness (nm)	50, 100, 200
Oxidation temperature (°C)	600, 650, 700, 750
Oxidation time (h)	2, 3, 4
Oxygen flow (SCCM)	1, 5, 10, 20
Argon flow (SCCM)	100

**Table 2:** Power law calibration coefficients and detection limits of TiO_2_ for ethanol and H_2_ at optimal working temperatures of 400 °C.

Gas	Detection Limit (ppm)	A	B
Ethanol	32	0.0405	0.9214
H_2_	90	0.0173	0.9029

**Table 3:** Summary of H_2_ and ethanol sensing performances of TiO_2_ NWs.

Synthesis Method	Composition	Gas	Gas Con: (ppm)	Temp: (°C)	RH%	Res (S)	Ref
Hydrothermal	Nano belts	Ethanol	400	350	40	13	[43]
Hydrothermal	Nanowires		1000	500	30	4.5	[44]
Electrospinning	Nanowires		1000	400	0	15.8	[45]
Hydrothermal	Nanowires		500	400	0	6	[46]
Hydrothermal	Nanotubes		47	500	0	15	[47]
Thermal oxidation	Nanowires		500	400	40	15	This work
VLS	Nanowires	Hydrogen	1000	500	0	1	[48]
Hydrothermal	Nanotubes		500	500	0	9.6	[47]
Anodic oxidation	Nanotubes		100	150	0	18	[49]
Hydrothermal	Nanorods		1000	200	0	0.87	[50]
Electrochemical Anodization	Nanotubes		20,000	25	0	0.25	[51]
Hydrothermal	Nanowires		5	RT	0	3.5	[52]
Thermal oxidation	Nanowires		500	400	40	5.2	This work
